# Under-reported aspects of diagnosis and treatment addressed in the Dutch-Flemish guideline for comprehensive diagnostics in disorders/differences of sex development

**DOI:** 10.1136/jmedgenet-2019-106354

**Published:** 2020-04-17

**Authors:** Yolande van Bever, Hennie T Brüggenwirth, Katja P Wolffenbuttel, Arianne B Dessens, Irene A L Groenenberg, Maarten F C M Knapen, Elfride De Baere, Martine Cools, Conny M A van Ravenswaaij-Arts, Birgit Sikkema-Raddatz, Hedi Claahsen-van der Grinten, Marlies Kempers, Tuula Rinne, Remko Hersmus, Leendert Looijenga, Sabine E Hannema

**Affiliations:** 1 Department of Clinical Genetics and DSD Expert Center Erasmus Medical Center, Erasmus Medical Center, Rotterdam, The Netherlands; 2 Department of Pediatric Urology and DSD Expert Center Erasmus Medical Center, Erasmus Medical Center, Rotterdam, The Netherlands; 3 Department of Child and Adolescent Psychiatry and DSD Expert Center Erasmus Medical Center, Erasmus Medical Center, Rotterdam, The Netherlands; 4 Department of Obstetrics and Prenatal Medicine and DSD Expert Center Erasmus Medical Center, Erasmus Medical Centre, Rotterdam, The Netherlands; 5 Center for Medical Genetics, University Hospital Ghent Center Medical Genetics, Ghent, Belgium; 6 Department of Internal Medicine and Paediatrics and Department of Pediatric Endocrinology, University Hospital Ghent, Ghent, Belgium; 7 Department of Genetics and DSD team, University Medical Center Groningen, Groningen, The Netherlands; 8 Department of Pediatric Endocrinology and DSD Expert Center Radboud UMC, Radboud University Medical Center, Amalia Children's Hospital, Nijmegen, The Netherlands; 9 Department of Clinical genetics and DSD Expert Center Radboud UMC, Radboud University Medical Center, Nijmegen, The Netherlands; 10 Department of Pathology, DSD Expert Center ErasmusMC, Erasmus MC-University Medical Centre, Rotterdam, The Netherlands; 11 Department of Pathology, Princess Máxima Center for Pediatric Oncology, Utrecht, The Netherlands; 12 Department of Pediatric Endocrinology and DSD Expert Center ErasmusMC, Erasmus Medical Center, 3015 GD Rotterdam, The Netherlands; 13 Department of Pediatrics, Leiden University Medical Center, Leiden, Zuid-Holland, The Netherlands

**Keywords:** DSD, NGS, guideline, diagnostic, prenatal

## Abstract

We present key points from the updated Dutch-Flemish guideline on comprehensive diagnostics in disorders/differences of sex development (DSD) that have not been widely addressed in the current (inter)national literature. These points are of interest to physicians working in DSD (expert) centres and to professionals who come across persons with a DSD but have no (or limited) experience in this area. The Dutch-Flemish guideline is based on internationally accepted principles. Recent initiatives striving for uniform high-quality care across Europe, and beyond, such as the completed COST action 1303 and the European Reference Network for rare endocrine conditions (EndoERN), have generated several excellent papers covering nearly all aspects of DSD. The Dutch-Flemish guideline follows these international consensus papers and covers a number of other topics relevant to daily practice. For instance, although next-generation sequencing (NGS)-based molecular diagnostics are becoming the gold standard for genetic evaluation, it can be difficult to prove variant causality or relate the genotype to the clinical presentation. Network formation and centralisation are essential to promote functional studies that assess the effects of genetic variants and to the correct histological assessment of gonadal material from DSD patients, as well as allowing for maximisation of expertise and possible cost reductions. The Dutch-Flemish guidelines uniquely address three aspects of DSD. First, we propose an algorithm for counselling and diagnostic evaluation when a DSD is suspected prenatally, a clinical situation that is becoming more common. Referral to ultrasound sonographers and obstetricians who are part of a DSD team is increasingly important here. Second, we pay special attention to healthcare professionals not working within a DSD centre as they are often the first to diagnose or suspect a DSD, but are not regularly exposed to DSDs and may have limited experience. Their thoughtful communication to patients, carers and colleagues, and the accessibility of protocols for first-line management and efficient referral are essential. Careful communication in the prenatal to neonatal period and the adolescent to adult transition are equally important and relatively under-reported in the literature. Third, we discuss the timing of (NGS-based) molecular diagnostics in the initial workup of new patients and in people with a diagnosis made solely on clinical grounds or those who had earlier genetic testing that is not compatible with current state-of-the-art diagnostics.

## Introduction

In recent years, many studies have been published on new diagnostic possibilities and management approaches in cohorts of patients suspected to have a disorder/difference of sex development (DSD).^1–13^ Based on these studies, it has become clear that services and institutions still differ in the composition of the multidisciplinary teams that provide care for patients who have a DSD.^11 14^ Several projects have now worked to resolve this variability in care. The European Cooperation in Science and Technology (EU COST) action BM1303 ‘A systematic elucidation of differences of sex development’ has been a platform to achieve European agreement on harmonisation of clinical management and laboratory practices.^15–17^ Another such initiative involved an update of the 2006 DSD consensus document by an international group of professionals and patient representatives.^18^ These initiatives have highlighted how cultural and financial aspects and the availability of resources differ significantly between countries and societies, a situation that hampers supranational agreement on common diagnostic protocols. As only a few national guidelines have been published in international journals, comparison of these guidelines is difficult even though such a comparison is necessary to capture the differences and initiate actions to overcome them. Nonetheless, four DSD (expert) centres located in the Netherlands and Flanders (the Dutch-speaking Northern part of Belgium) have collaborated to produce a detailed guideline on diagnostics in DSD.^19^ This shows that a supranational guideline can be a reasonable approach for countries with similarly structured healthcare systems and similar resources. Within the guideline there is agreement that optimisation of expertise and care can be achieved through centralisation, for example, by limiting analysis of next-generation sequencing (NGS)-based diagnostic panels to only a few centres and by centralising pathological review of gonadal tissues. International networks such as the European Reference Network for rare endocrine conditions (EndoERN), in which DSD is embedded, may facilitate the expansion of this kind of collaboration across Europe.

This paper highlights key discussion points in the Dutch-Flemish guideline that have been insufficiently addressed in the literature thus far because they reflect evolving technologies or less visible stakeholders. For example, prenatal observation of an atypical aspect of the genitalia indicating a possible DSD is becoming increasingly common, and we discuss appropriate counselling and a diagnostic approach for these cases, including the option of using NGS-based genetic testing. So far, little attention has been paid to this process.^20 21^ Furthermore, informing patients and/or their parents about atypical sex development and why this may warrant referral to a specialised team may be challenging, especially for professionals with limited experience in DSD.^22 23^ Therefore, a section of the Dutch-Flemish guideline was written for these healthcare providers. Moreover, this enables DSD specialists to refer to the guideline when advising a referral. Transition from the prenatal to the postnatal team and from the paediatric to the adult team requires optimal communication between the specialists involved. Application of NGS-based techniques may lead to a higher diagnostic yield, providing a molecular genetic diagnosis in previously unsolved cases.^16^ We address the timing of this testing and the problems associated with this technique such as the interpretation of variants of unknown clinical significance (VUS). Similarly, histopathological interpretation and classification of removed gonadal tissue is challenging and would benefit from international collaboration and centralisation of expertise.

## Methods

For the guideline revision, an interdisciplinary multicentre group was formed with all members responsible for updating the literature for a specific part of the guideline. Literature search in PubMed was not systematic, but rather intended to be broad in order to cover all areas and follow expert opinions. This approach is more in line with the Clinical Practice Advisory Document method described by Burke *et al* 24 for guidelines involving genetic practice because it is often troublesome to substantiate such guidelines with sufficient evidence due to the rapid changes in testing methods, for example, gene panels. All input provided by the group was synthesised by the chairperson (YvB), who also reviewed abstracts of papers on DSD published between 2010 and September 2017 for the guideline and up to October 2019 for this paper. Abstracts had to be written in English and were identified using a broad range of Medical Subject Headings terms (eg, DSD, genetic, review, diagnosis, diagnostics, 46, XX DSD, 46, XY DSD, guideline, multidisciplinary care). Next, potentially relevant papers on diagnostic procedures in DSD were selected. Case reports were excluded, as were articles that were not open access or retrievable through institutional access. Based on this, a draft guideline was produced that was in line with the international principles of good diagnostic care in DSD. This draft was discussed by the writing committee and, after having obtained agreement on remaining points of discussion, revised into a final draft. This version was sent to a broad group of professionals from academic centres and DSD teams whose members had volunteered to review the draft guideline. After receiving and incorporating their input, the final version was presented to the paediatric and genetic associations for approval. After approval by the members of the paediatric (NVK), clinical genetic (VKGN) and genetic laboratory (VKGL) associations, the guideline was published on their respective websites.^19^ Although Turner syndrome and Klinefelter syndrome are considered to be part of the DSD spectrum, they are not extensively discussed in this diagnostic guideline as guidelines dedicated to these syndromes already exist.^25 26^ However, some individuals with Turner syndrome or Klinefelter syndrome may present with ambiguous or atypical genitalia and may therefore initially follow the DSD diagnostic process.

## Guideline highlights

### Prenatal setting

#### Presentation

The most frequent prenatal presentation of a DSD condition is atypical genitalia found on prenatal ultrasound as an isolated finding or in combination with other structural anomalies. This usually occurs after the 20-week routine medical ultrasound for screening of congenital anomalies, but may also occur earlier, for example, when a commercial ultrasound is performed at the request of the parents.

Another way DSD can be diagnosed before birth is when invasive prenatal genetic testing carried out for a different reason, for example, due to suspicion of other structural anomalies, reveals a discrepancy between the genotypic sex and the phenotypic sex seen by ultrasound. In certified laboratories, the possibility of a sample switch is extremely low but should be ruled out immediately. More often, the discrepancy will be due to sex-chromosome mosaicism or a true form of DSD.

A situation now occurring with increasing frequency is a discrepancy between the genotypic sex revealed by non-invasive prenatal testing (NIPT), which is now available to high-risk pregnant women in the Netherlands and to all pregnant women in Belgium, and later ultrasound findings. NIPT screens for CNVs in the fetus. However, depending on legal restrictions and/or ethical considerations, the X and Y chromosomes are not always included in NIPT analysis and reports. If the X and Y chromosomes are included, it is important to realise that the presence of a Y-chromosome does not necessarily imply male fetal development. At the time that NIPT is performed (usually 11–13 weeks), genital development cannot be reliably appreciated by ultrasound, so any discrepancy or atypical aspect of the genitalia will only be noticed later in pregnancy and should prompt further evaluation.

#### Counselling and diagnostics

If a DSD is suspected, first-line sonographers and obstetricians should refer the couple to their colleague prenatal specialists working with or in a DSD team. After confirming an atypical genital on ultrasound, the specialist team should offer the couple a referral for genetic counselling to discuss the possibility of performing invasive prenatal testing (usually an amniocentesis) to identify an underlying cause that fits the ultrasound findings.^22 23^ To enable the parents to make a well-informed decision, prenatal counselling should, in our opinion, include: information on the ultrasound findings and the limitations of this technique; the procedure(s) that can be followed, including the risks associated with an amniocentesis; and the type of information genetic testing can and cannot provide. Knowing which information has been provided and what words have been used by the prenatal specialist is very helpful for those involved in postnatal care.

It is important that parents understand that the biological sex of a baby is determined by a complex interplay of chromosomes, genes and hormones, and thus that assessment of the presence or absence of a Y-chromosome alone is insufficient to assign the sex of their unborn child or, as in any unborn child, say anything about the child’s future gender identity.

Expecting parents can be counselled by the clinical geneticist and the psychologist from the DSD team, although other DSD specialists can also be involved. The clinical geneticist should be experienced in prenatal counselling and well informed about the diagnostic possibilities given the limited time span in which test results need to be available to allow parents to make a well-informed decision about whether or not to continue the pregnancy. Termination of pregnancy can be considered, for instance, in a syndromic form of DSD with multiple malformations, but when the DSD occurs as an apparently isolated condition, expecting parents may also consider termination of pregnancy, which, although considered controversial by some, is legal in Belgium and the Netherlands. The psychologist of the DSD team can support parents during and after pregnancy and help them cope with feelings of uncertainty and eventual considerations of a termination of pregnancy, as well as with practical issues, for example, how to inform others. The stress of not knowing exactly what the child’s genitalia will look like and uncertainty about the diagnosis, treatment and prognosis cannot be avoided completely. Parents are informed that if the postnatal phenotype is different from what was prenatally expected, the advice given about diagnostic testing can be adjusted accordingly, for example, if a hypospadias is milder than was expected based on prenatal ultrasound images. In our experience, parents appreciate having already spoken to some members of the DSD team during pregnancy and having a contact person before birth.

After expert prenatal counselling, a significant number of pregnant couples decline prenatal testing (personal experience IALG, MK, ABD, YvB, MC and HC-vdG). At birth, umbilical cord blood is a good source for (molecular) karyotyping and storage of DNA and can be obtained by the obstetrician, midwife or neonatologist. The terminology used in communication with parents should be carefully chosen,^22 23^ and midwives and staff of neonatal and delivery units should be clearly instructed to use gender-neutral and non-stigmatising vocabulary (eg, ‘your baby’) as long as sex assignment is pending.

**Figure 1:**
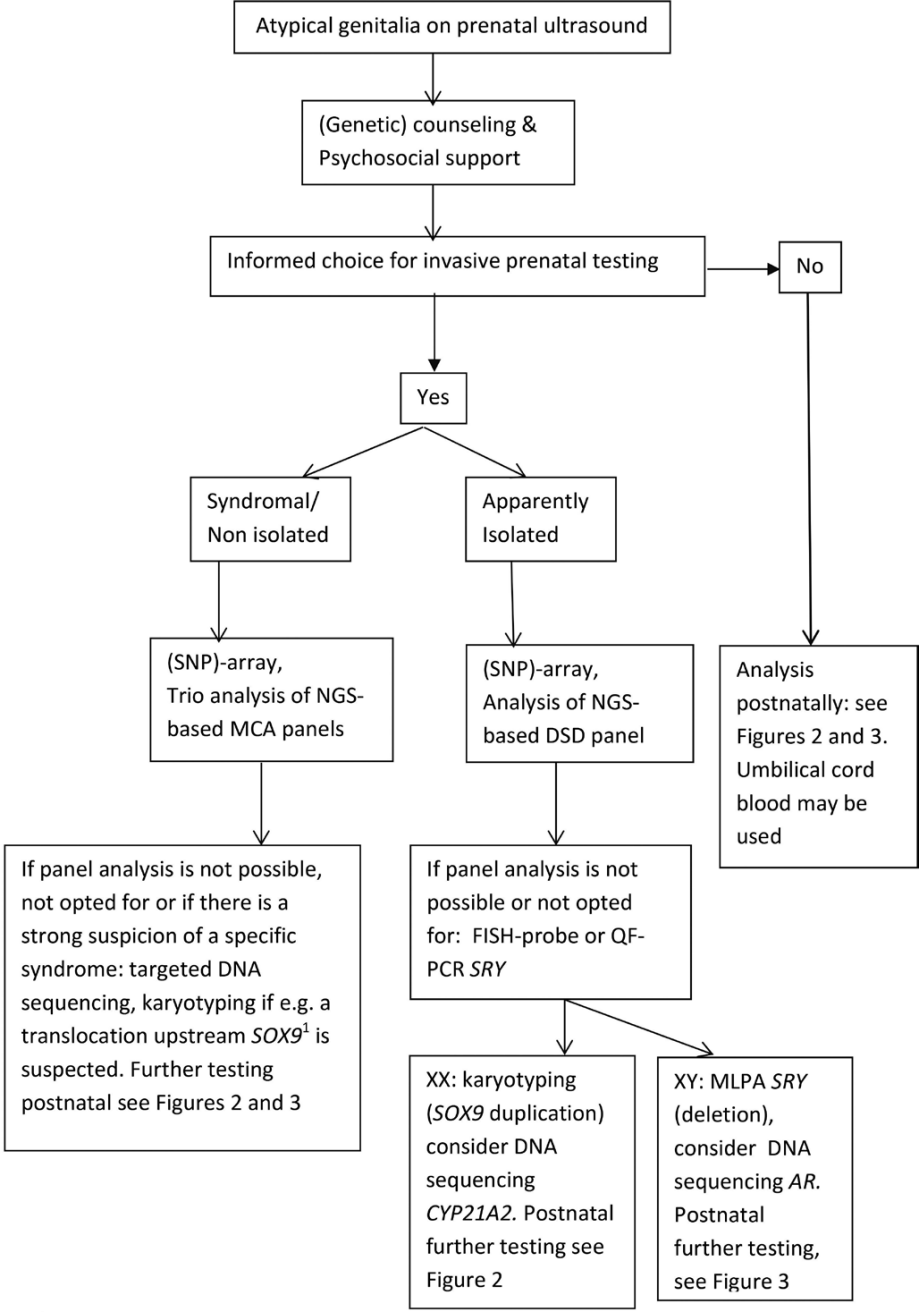
Disorders/differences of sex development (DSD) in the prenatal setting: a diagnostic algorithm. *SOX9: upstream anomalies and balanced translocations at promotor sites! Conventional karyotyping can be useful. NGS, next-generation sequencing.

An algorithm for diagnostic evaluation of a suspected DSD in the prenatal situation is proposed in figure 1. When couples opt for invasive prenatal diagnosis, the genetic analysis usually involves an (SNP)-array. It was recently estimated that >30% of individuals who have a DSD have additional structural anomalies, with cardiac and neurological anomalies and fetal growth restriction being particularly common.^27 28^ If additional anomalies are seen, the geneticist can consider specific gene defects that may underlie a known genetic syndrome or carry out NGS. NGS-based techniques have also now made their appearance in prenatal diagnosis of congenital anomalies.^29 30^ Panels using these techniques can be specific for genes involved in DSD, or be larger panels covering multiple congenital anomalies, and are usually employed with trio-analysis to compare variants identified in the child with the parents’ genetics.^29–31^ Finding a genetic cause before delivery can help reduce parental stress in the neonatal period and speed up decisions regarding gender assignment. In such cases there is no tight time limit, and we propose completing the analysis well before the expected delivery.

### First contact by a professional less experienced in DSD

Whereas most current guidelines start from the point when an individual has been referred to the DSD team,^1 15^ the Dutch-Flemish guideline dedicates a chapter to healthcare professionals less experienced in DSD as they are often the first to suspect or identify such a condition. Apart from the paper of Indyk,^7^ little guidance is available for these professionals about how to act in such a situation. The chapter in the Dutch-Flemish guideline summarises the various clinical presentations that a DSD can have and provides information on how to communicate with parents and/or patients about the findings of the physical examination, the first-line investigations and the need for prompt referral to a specialised centre for further evaluation. Clinical examples are offered to illustrate some of these recurring situations. The medical issues in DSD can be very challenging, and the social and psychological impact is high. For neonates with ambiguous genitalia, sex assignment is an urgent and crucial issue, and it is mandatory that parents are informed that it is possible to postpone registration of their child’s sex. In cases where sex assignment has already taken place, the message that the development of the gonads or genitalia is still atypical is complicated and distressing for patients and parents or carers. A list of contact details for DSD centres and patient organisations in the Netherlands and Flanders is attached to the Dutch-Flemish guideline. Publishing such a list, either in guidelines or online, can help healthcare professionals find the nearest centres for consultations and provide patients and patient organisations with an overview of the centres where expertise is available.

### Timing and place of genetic testing using NGS-based gene panels

**Figure 2:**
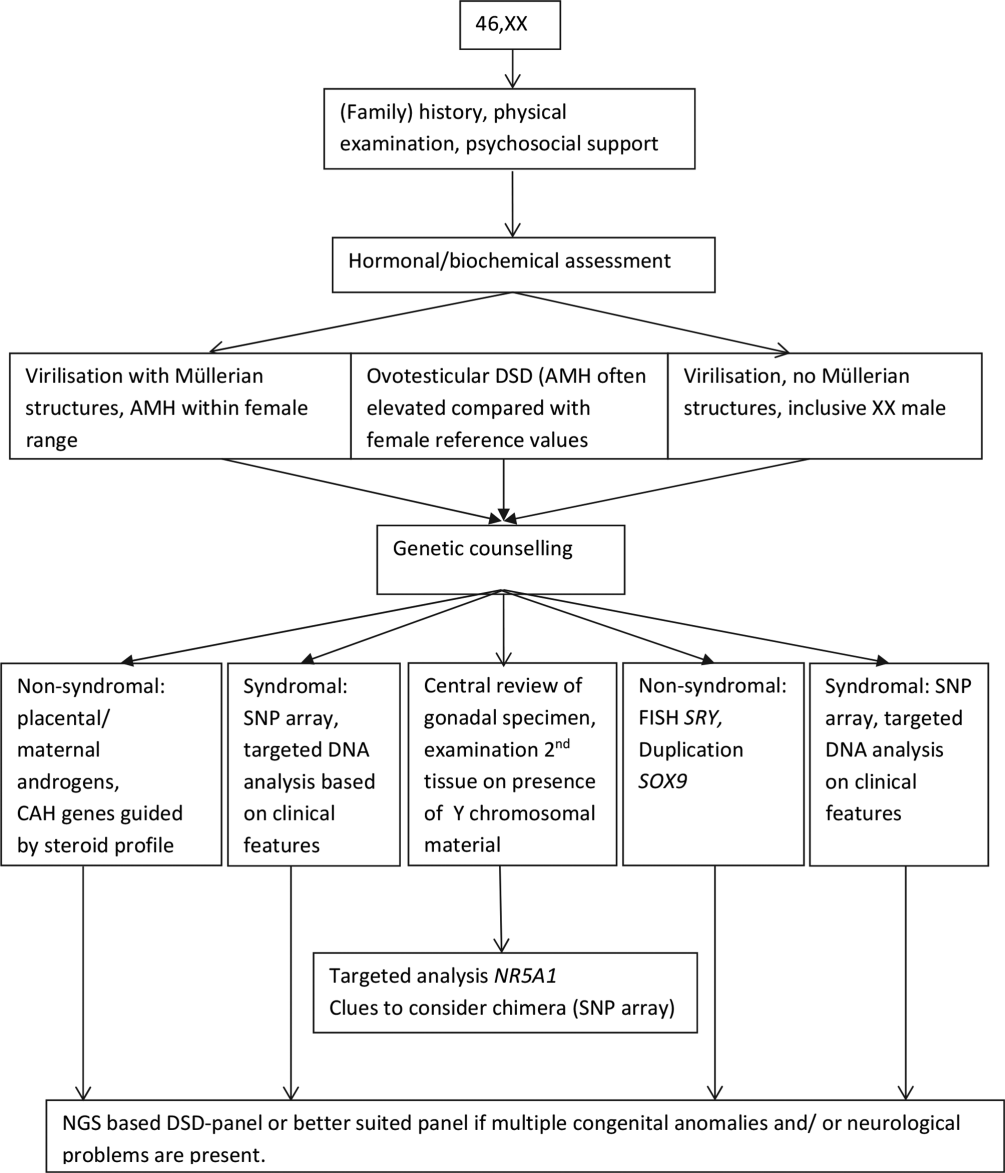
46, XX disorders/differences of sex development (DSD) in the postnatal setting: a diagnostic algorithm. NGS, next-generation sequencing. CAH, Congenital adrenal hyperplasia; AMH, Anti-Müllerian Hormone.

**Figure 3:**
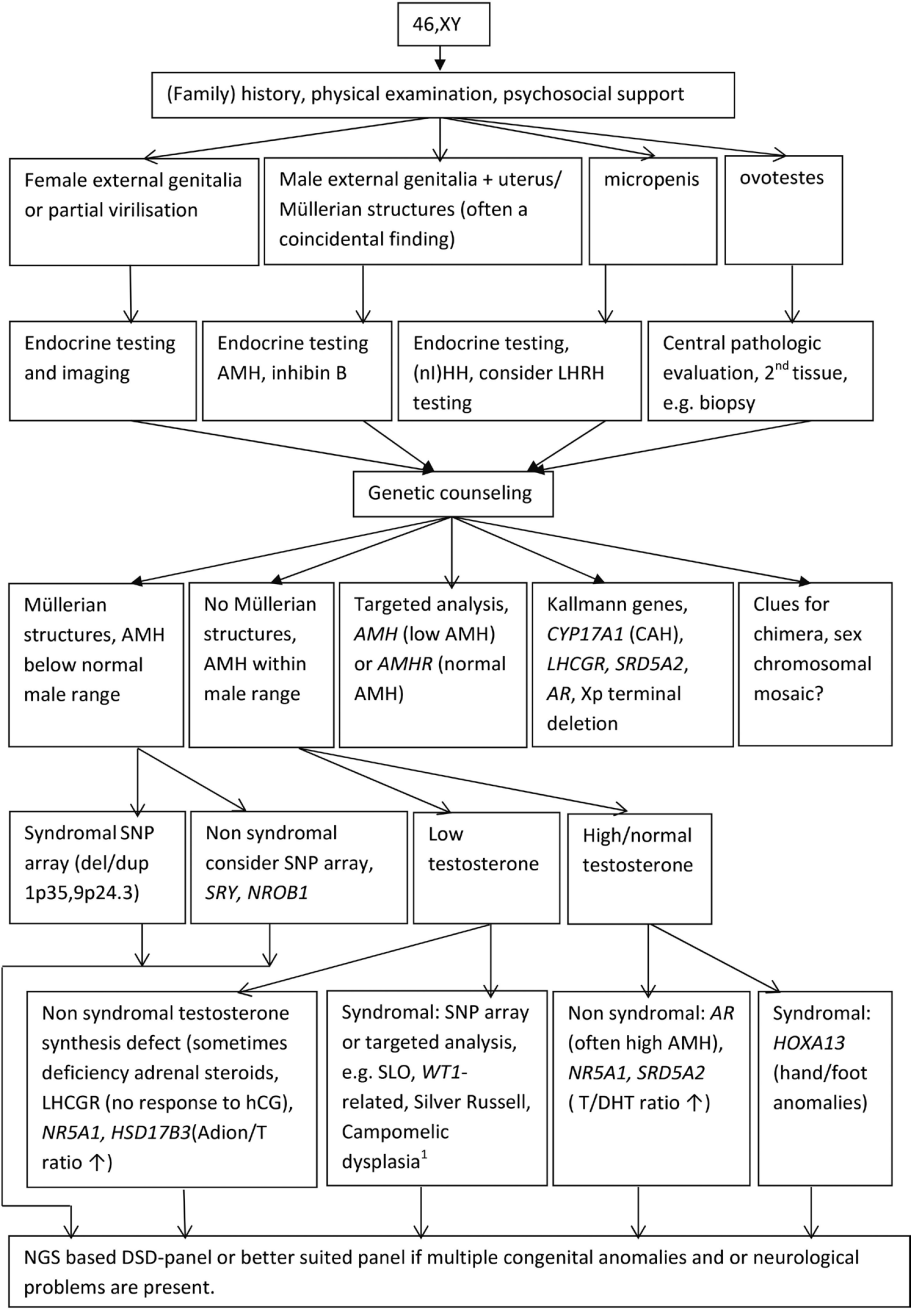
46, XY disorders/differences of sex development (DSD) in the postnatal setting: a diagnostic algorithm. ^*^ *SOX9:* upstream anomalies and balanced translocations at promotor sites! Conventional karyotyping can be useful. NGS, next-generation sequencing.

The diagnostic workup that is proposed for 46, XX and 46, XY DSD is shown in figures 2 and 3, respectively. Even with the rapidly expanding molecular possibilities, a (family) history and a physical examination remain the essential first steps in the diagnostic process. Biochemical and hormonal screening aim at investigating serum electrolytes, renal function and the hypothalamic-pituitary-gonadal and hypothalamic-pituitary-adrenal axes. Ultrasound screening of kidneys and internal genitalia, as well as establishing genotypic sex, should be accomplished within 48 hours and complete the baseline diagnostic work-up of a child born with ambiguous genitalia.^1 16 32 33^

Very recently, a European position paper has been published focusing on the genetic workup of DSD.^16^ It highlights the limitations and drawbacks of NGS-based tests, which include the chance of missing subtle structural variants such as CNVs and mosaicism and the fact that NGS cannot detect methylation defects or other epigenetic changes.^16 28 31^ Targeted DNA analysis is preferred in cases where hormonal investigations suggest a block in steroidogenesis (eg, 11-β-hydroxylase deficiency, 21-hydroxylase deficiency), or in the context of a specific clinical constellation such as the often coincidental finding of Müllerian structures in a boy with normal external genitalia or cryptorchidism, that is, persistent Müllerian duct syndrome.^33 34^ Alternative tests should also be considered depending on the available information. Sometimes, a simple mouth swab for FISH analysis can detect mosaic XY/X in a male with hypospadias or asymmetric gonadal development or in a female with little or no Turner syndrome stigmata and a normal male molecular karyotyping profile or peripheral blood karyotype. Such targeted testing avoids incidental findings and is cheaper and faster than analysis of a large NGS-based panel, although the cost difference is rapidly declining.

However, due to the genetic and phenotypic heterogeneity of DSD conditions, the most cost-effective next steps in the majority of cases are whole exome sequencing followed by panel analysis of genes involved in genital development and function or trio-analysis of a large gene panel (such as a Mendeliome).^16 35–38^ Pretest genetic counselling involves discussing what kind of information will be reported to patients or parents and the chance of detecting VUS, and the small risk of incidental findings when analysing a DSD panel should be mentioned. Laboratories also differ in what class of variants they report.^39^ In our experience, the fear of incidental findings is a major reason why some parents refrain from genetic testing.

Timing of the DSD gene panel analysis is also important. While some patients or parents prefer that all diagnostic procedures be performed as soon as possible, others need time to reflect on the complex information related to more extensive genetic testing and on its possible consequences. If parents or patients do not consent to panel-based genetic testing, analysis of specific genes, such as *WT1,* should be considered when appropriate in view of the clinical consequences if a mutation is present (eg, clinical surveillance of renal function and screening for Wilms’ tumour in the case of *WT1* mutations). Genes that are more frequently involved in DSD (eg, *SRY, NR5A1*) and that match the specific clinical and hormonal features in a given patient could also be considered for sequencing. Targeted gene analysis may also be preferred in centres located in countries that do not have the resources or technical requirements to perform NGS panel-based genetic testing. Alternatively, participation by these centres in international collaborative networks may allow them to outsource the molecular genetic workup abroad.

Gene panels differ between centres and are regularly updated based on scientific progress. A comparison of DSD gene panels used in recent studies can be found at https://www.nature.com/articles/s41574-018-0010-8%23Sec46.^15^ The panels currently used at the coauthors’ institutions can be found on their respective websites. Given the pace of change, it is important to regularly consider repeating analysis in patients with an unexplained DSD, for example, when they transition into adult care or when they move from one centre to another. This also applies to patients in whom a clinical diagnosis has never been genetically confirmed. Confusion may arise when the diagnosis cannot be confirmed or when a mutation is identified in a different gene, for example, *NR5A1* in someone with a clinical diagnosis of CAIS that has other consequences for relatives. Hence, new genetic counselling should always accompany new diagnostic endeavours.

### Class 3 variants and histopathological examinations

The rapidly evolving diagnostic possibilities raise new questions: What do laboratories report? How should we deal with the frequent findings of mainly unique VUS or class 3 variants (ACMG recommendation) in the many different DSD-related genes in the diagnostic setting? Reporting VUS can be a source of uncertainty for parents, but not reporting these variants precludes further investigations to determine their possible pathogenicity. It can also be difficult to prove variant pathogenicity, both on gene-level and variant-level.^39^ Moreover, given the gonad-specific expression of some genes and the variable phenotypic spectrum and reduced penetrance, segregation analysis is not always informative. A class 3 variant that does not fit the clinical presentation may be unrelated to the observed phenotype, but it could also represent a newly emerging phenotype. This was recently demonstrated by the identification of the *NR5A1* mutation, R92W, in individuals with 46, XX testicular and ovotesticular DSD.^40^ This gene had previously been associated with 46, XY DSD. In diagnostic laboratories, there is usually no capacity or expertise to conduct large-scale functional studies to determine pathogenicity of these unique class 3 VUS in the different genes involved in DSD. Functional validation of variants identified in novel genes may be more attractive in a research context. However, for individual families with VUS in well-established DSD genes such as *AR* or *HSD17B3,* functional analysis may provide a confirmed diagnosis that implies for relatives the option of undergoing their own DNA analysis and estimating the genetic risk of their own future offspring. This makes genetic follow-up important in these cases and demonstrates the usefulness of international databases and networks and the centralisation of functional studies of genetic variants in order to reduce costs and maximise expertise.

The same is true for histopathological description, germ-cell tumour risk assessment in specific forms of DSD and classification of gonadal samples. Germ-cell tumour risk is related to the type of DSD (among other factors), but it is impossible to make risk estimates in individual cases.^41–44^ Gonadectomy may be indicated in cases with high-risk dysgenetic abdominal gonads that cannot be brought into a stable superficial (ie, inguinal, labioscrotal) position that allows clinical or radiological surveillance, or to avoid virilisation due to 5-alpha reductase deficiency in a 46, XY girl with a stable female gender identity.^45^ Pathological examination of DSD gonads requires specific expertise. For example, the differentiation between benign germ cell abnormalities, such as delayed maturation and (pre)malignant development of germ cells, is crucial for clinical management but can be very troublesome.^46^ Centralised pathological examination of gonadal biopsy and gonadectomy samples in one centre, or a restricted number of centres, on a national scale can help to overcome the problem of non-uniform classification and has proven feasible in the Netherlands and Belgium. We therefore believe that uniform assessment and classification of gonadal differentiation patterns should also be addressed in guidelines on DSD management.

International databases of gonadal tissues are crucial for learning more about the risk of malignancy in different forms of DSD, but they are only reliable if uniform criteria for histological classification are strictly applied.^46^ These criteria could be incorporated in many existing networks such as the I-DSD consortium, the Disorders of Sex Development Translational Research Network, the European Reference Network on Urogenital Diseases (eUROGEN), the EndoERN and COST actions.^15–17 47^

### Communication at the transition from paediatric to adult care

Paediatric and adult teams need to collaborate closely to facilitate a well-organised transition from paediatric to adult specialist care.^15 48–50^ Both teams need to exchange information optimally and should consider transition as a longitudinal process rather than a fixed moment in time. Age-appropriate information is key at all ages, and an overview of topics to be discussed at each stage is described by Cools *et al*.^15^ Table 1 shows an example of how transition can be organised.

**Table 1:** Example of transition table as used in the DSD clinic of the Erasmus Medical Center

Age (years)	Endocrine, urologic, genetic, psychosocial care multidisciplinary team
<11	Diagnostics, follow-up, treatment as indicated for the specific DSD. Provide information about the condition to parents and children, including information about the expected pubertal changes. Obtain informed consent whenever needed from parents as well as informed assent from children, in compliance with the Dutch and Belgian Law. Support children and parents to promote coping. Inform the children about their condition, taking into account their developmental phase. Observe the children’s developing gender identity and facilitate open discussion if needed.
11–12	Evaluate puberty. Hormonal testing and treatment if needed. Provide information. Imaging studies as necessary. If applicable, in case of a girl discuss future gonadal surgeries or vaginal dilatation depending on sexual development. Provide information on pros and cons. Evaluate if genetic tests should be expanded and if parents and children are open to this. Provide the children with additional and more detailed information on diagnosis and consequences for pubertal development. Establish if parents/carers need help with informing the children.
11–17	Multidisciplinary team: preparation for adolescence and adulthood. Responsibility shifting from parents to patients (eg, knowledge about diagnosis and practical management of medication, independent doctor visits, where to find information, what help is available). Check that adolescents are fully informed about diagnosis, management, follow-up and both necessary and optional diagnostic and therapeutic procedures. Sexual development, romantic relationships, sex education, optimal procedures to facilitate sexual intercourse like vaginal dilation and fertility (options) need to be addressed. This age period may not be the best time to perform broad genetic analysis as adolescents are often not yet be able to process the consequences of uncertain or incidental findings. However, if genetic testing is performed, consent should be obtained from the adolescents as well.
17–18	Transfer of care with adult and paediatric specialists in a joint multidisciplinary meeting and/or outpatient clinical setting.

Ages are approximate, and cognitive, physical and socioemotional development should be taken into account. The process of informing children about their condition and making them more and more responsible for the management is gradual.

DSD, disorders/differences of sex development.

Psychological support and the continued provision of information remains important for individuals with a DSD at all ages.^15 22^ In addition to the information given by the DSD team members, families and patients can benefit from resources such as support groups and information available on the internet.^47^ Information available online should be checked for accuracy and completeness when referring patients and parents to internet sites.

## Recommendations for future actions

Most guidelines and articles on the diagnosis and management of DSD are aimed at specialists and are only published in specialist journals or on websites for endocrinologists, urologists or geneticists. Yet there is a need for guidelines directed towards first-line and second-line healthcare workers that summarise the recommendations about the first crucial steps in the management of DSD. These should be published in widely available general medical journals and online, along with a national list of DSD centres. Furthermore, DSD (expert) centres should provide continuous education to all those who may be involved in the identification of individuals with a DSD in order to enable these healthcare professionals to recognise atypical genitalia, to promptly refer individuals who have a DSD and to inform the patient and parents about this and subsequent diagnostic procedures.

As DSD continues to be a rare condition, it will take time to evaluate the effects of having such a guideline on the preparedness of first-line and second-line healthcare workers to recognise DSD conditions. One way to evaluate this might be the development and use of questionnaires asking patients, carers and families and referring physicians how satisfied they were with the initial medical consultation and referral and what could be improved. A helpful addition to existing international databases that collect information on genetic variations would be a list of centres that offer suitable functional studies for certain genes, ideally covering the most frequently mutated genes (at minimum).

Patient organisations can also play an important role in informing patients about newly available diagnostic or therapeutic strategies and options, and their influence and specific role has now been recognised and discussed in several publications.^17 47^ However, it should be kept in mind that these organisations do not represent all patients, as a substantial number of patients and parents are not member of such an organisation.

Professionals have to provide optimal medical care based on well-established evidence, or at least on broad consensus. Yet not everything can be regulated by recommendations and guidelines. Options, ideas and wishes should be openly discussed between professionals, patients and families within their confidential relationship. This will enable highly individualised holistic care tailored to the patient’s needs and expectations. Once they are well-informed of all available options, parents and/or patients can choose what they consider the optimal care for their children or themselves.^15 16^

## Conclusion

The Dutch-Flemish guideline uniquely addresses some topics that are under-represented in the literature, thus adding some key aspects to those addressed in recent consensus papers and guidelines.^15–17 33 47^

As more children with a DSD are now being identified prenatally, and the literature on prenatal diagnosis of DSD remains scarce,^20 21^ we propose a prenatal diagnostic algorithm and emphasise the importance of having a prenatal specialist involved in or collaborating with DSD (expert) centres.

We also stress that good communication between all involved parties is essential. Professionals should be well informed about protocols and communication. Collaboration between centres is necessary to optimise aspects of care such as uniform interpretation of gonadal pathology and functional testing of class 3 variants found by genetic testing. Guidelines can provide a framework within which individualised patient care should be discussed with all stakeholders.
